# Biocontrol potential of *Bacillus velezensis* EM-1 associated with suppressive rhizosphere soil microbes against tobacco bacterial wilt

**DOI:** 10.3389/fmicb.2022.940156

**Published:** 2022-08-23

**Authors:** Xiaona Sui, Xiaobin Han, Jianmin Cao, Yiqiang Li, Yuan Yuan, Jianyu Gou, Yanfen Zheng, Chen Meng, Chengsheng Zhang

**Affiliations:** ^1^Pest Integrated Management Key Laboratory of China Tobacco, Tobacco Research Institute of Chinese Academy of Agricultural Sciences, Qingdao, China; ^2^Biological Organic Fertilizer Engineering Technology Center of China Tobacco, Zunyi Branch of Guizhou Tobacco Company, Zunyi, China

**Keywords:** *Ralstonia solanacearum*, biocontrol agent, volatile compounds, lipopeptides, polyketides, induced resistance

## Abstract

Tobacco bacterial wilt caused by *Ralstonia solanacearum* is one of the most devastating diseases. Microbial keystone taxa were proposed as promising targets in plant disease control. In this study, we obtained an antagonistic *Bacillus* isolate EM-1 from bacterial wilt-suppressive soil, and it was considered rhizosphere-resident bacteria based on high (100%) 16S rRNA gene similarity to sequences derived from high-throughput amplicon sequencing. According to 16S rRNA gene sequencing and MLSA, strain EM-1 was identified as *Bacillus velezensis*. This strain could inhibit the growth of *R. solanacearum*, reduce the colonization of *R. solanacearum* in tobacco roots, and decrease the incidence of bacterial wilt disease. In addition, strain EM-1 also showed a strong inhibitory effect on other phytopathogens, such as *Alternaria alternata* and *Phytophthora nicotianae*, indicating a wide antagonistic spectrum. The antimicrobial ability of EM-1 can be attributed to its volatile, lipopeptide and polyketide metabolites. Iturin A (C14, C15, and C16) was the main lipopeptide, and macrolactin A and macrolactin W were the main polyketides in the fermentation broth of EM-1, while heptanone and its derivatives were dominant among the volatile organic compounds. Among them, heptanones and macrolactins, but not iturins, might be the main potential antibacterial substances. Complete genome sequencing was performed, and the biosynthetic gene clusters responsible for iturin A and macrolactin were identified. Moreover, strain EM-1 can also induce plant resistance by increasing the activity of CAT and PPO in tobacco. These results indicated that EM-1 can serve as a biocontrol *Bacillus* strain for tobacco bacterial wilt control. This study provides a better insight into the strategy of exploring biocontrol agent based on rhizosphere microbiome.

## Introduction

*Ralstonia solanacearum* (*Rs*) is a soilborne Gram-negative bacterium that can cause disastrous bacterial wilt (BW) in various plant species, for example, tobacco, tomato, potato, banana, eggplant, and pepper (Cunnac et al., 2004). Bacterial pathogens infect plant roots through wounds or natural openings, produce excessive extracellular polysaccharides (EPS) within the vascular system, and cause wilt, ultimately leading to the death of severely infected plants (Genin, 2010). BW mainly occurs at the maturity stage of tobacco and thus lead to huge economic losses. Due to its genetic diversity and wide geographic distribution, *Rs* presents a rather complex life cycle and can survive in both soil and water for years (Jiang et al., 2017). Currently, chemical fungicides dominated by antibiotics and inorganic copper-based pesticides are used for bacterial wilt control. However, the use of antibiotics in plant protection is increasingly restricted, and the long-term use of cupric pesticides negatively affects the environment. Therefore, there is an urgent need to find a novel and effective prevention strategy for bacterial wilt control.

Antagonistic microorganisms have been recognized as effective and sustainable for controlling plant diseases. To date, a large number of antagonistic microbes against *Rs* have been identified, including *Bacillus* spp. (Gao et al., 2013), *Pseudomonas* spp. (Maji and Chakrabartty, 2014), *Trichoderma* spp. (Mohamed et al., 2020), and *Streptomyces* spp. (Ling et al., 2020). Among them, the genus *Bacillus* has many advantages, including fast reproduction, strong survival ability, and long storage period. Moreover, *Bacillus* species can produce a wide range of antibiotic compounds, including cyclic lipopeptides (CLPS) and antifungal proteins, which play an important role in plant pathogen inhibition (Ongena and Jacques, 2008; Kwon and Kim, 2014). Therefore, bacteria of the genus *Bacillus* are considered as an ideal biocontrol agent and have been widely researched and used.

A large number of *Bacillus* strains have been screened for controlling tobacco bacterial wilt (Wu et al., 2016; Tahir et al., 2017; Agarwal et al., 2020). However, these strains were isolated mainly based on the interaction between individual microbial strains and plants, resulting in poor field colonization ability and unstable biocontrol effects (Lebeis, 2014). The latest research shows that a few key microorganisms, called “core microbiome,” may play the most important functions in the microbial community (Banerjee et al., 2018); thus, the microbial keystone taxa have been proposed as promising targets in plant disease control (Agler et al., 2016). Qi et al. (2019) reported that healthy soil harbors more key microbes (e.g., *Bacillus* and *Actinobacteria*) than bacterial wilt-susceptible soil. Our previous study analyzed the microbial community structure and function of tobacco bacterial wilt-suppressive soil and found that *Pseudomonas* was enriched in suppressive soil, which displayed strong inhibitory effects on *R. solanacearum* (Zheng et al., 2021). The current study aims to screen biocontrol *Bacillus* strains from bacterial wilt-suppressive soil and to evaluate their inhibitory efficacy against tobacco bacterial wilt. The complete genome of the selected strain was analyzed to reveal the possible mechanisms of disease control. The results will provide a better insight into the strategy of exploring biocontrol agent based on rhizosphere microbiome.

## Materials and methods

### Microbial and plant resources

*R. solanacearum* RS10 was isolated from a diseased tobacco plant (Bai et al., 2016). The phytopathogenic fungi used in this study were *Alternaria alternata, Phytophthora nicotianae, Botrytis cinerea, Fusarium oxysporum, Cucumber anthracnose*, and *Rhizoctonia cerealis* (Xie et al., 2021; Wang et al., 2022). All strains were preserved in our laboratory and stored in 25% (v/v) glycerol at −80°C.

Seeds of tobacco (*Nicotiana tabacum* cv Xiaohuangjin 1025) were sown in sterilized soil (volume of soil:vermiculite, 3:1) and grown in a greenhouse with a 16-h light/8-h dark cycle, day and night temperatures of 29 and 28°C, respectively, and a relative humidity of 70–80%.

All the phytopathogenic fungi were cultured on potato dextrose agar (PDA, 6 g potato infusion, 20 g dextrose, 15 g agar, distilled water 1 L) medium, except *P. nicotianae*, which was cultured on oatmeal agar (OA, oatmeal 30 g, agar 15 g, distilled water 1 L) medium. *R. solanacearum* RS10 was cultured in nutrient broth (NB, 10 g glucose, 5 g tryptone, 0.5 g yeast extract, 3 g beef extract, distilled water 1 L, pH = 7.0) medium or nutrient agar (NA, NB medium containing an additional 15 g agar per liter) medium. Unless otherwise stated, the isolate EM-1 was cultured on Luria–Bertani (LB, 10 g tryptone, 5 g yeast extract, 10 g sodium chloride, distilled water 1 L) medium.

### Screening of candidate biocontrol *Bacillus* isolates

According to 16S rRNA gene amplicon sequencing data from our previous study (Zheng et al., 2021), an OTU-based analysis was used to analyze the relative abundance of *Bacillus*. The bacteria were isolated according to Khanh et al. (2020). The biocontrol microbes were selected from a total of 43 *Bacillus* isolates by using the confrontation culture method (Chen et al., 2020). Specifically, *Rs* and *Bacillus* isolates were grown for 18–24 h to OD_600_ = 0.8–1.0 in NB medium at 28°C in a shaking incubator (180 rpm). *Rs* (0.2 ml) was included in NA solid medium (20 ml) to make plates, which were used to screen biocontrol *Bacillus* strains. The suspensions of *Bacillus* isolates (4 μl) were point-inoculated on the plates containing *Rs*. Sterile distilled water was used as a control. After incubation at 28°C for 48 h, the bacterial inhibition ability was estimated by measuring the inhibition zone diameter. Based on preliminary experimental results, the isolate EM-1 was selected for further investigation.

### Antagonistic activity of the isolate EM-1 *in vitro*

The confrontation culture method was used for antifungal activity determination (Kurniawan et al., 2018). An agar block (5 mm in diameter) from the margin of 5-day-old culture of the fungal pathogen was placed in the center of the PDA medium. The strain EM-1 was grown for 18–24 h to OD_600_ = 1.0 in NB medium at 28°C in a shaking incubator (180 rpm), and 4 μl of EM-1 suspensions were point-inoculated on both sides of the Petri dish. Equal volumes of sterile distilled water served as control. The plates were then incubated at 28°C. The diameter of the fungal colony was measured after the control culture had covered the plate. The percentage of mycelial growth inhibition was calculated using the following equation:


$$\begin{array}{l} \text{ Percentage inhibition}(\%)=\;[(\text{control colony diameter}\\ -\text{ treated colony diameter})\;/\text{ control colony diameter}]\;\times \;100 \end{array}$$

The fumigation effect of EM-1 on *Rs* was tested according to You et al. (2015). Suspensions (20 μl) of *Rs* and EM-1 were spread evenly on different NA medium plates, respectively, and two plates (the plate inoculated with *Rs* was upper) were sealed together with polyethylene (PE) stretch-wrap. After incubation at 28°C for 24 h, the growth of *Rs* was observed.

### Identification of the isolate EM-1

Genomic DNA of the strain EM-1 was isolated using the Solarbio DNA Extraction Kit (Solarbio, Beijing, China). 16S rRNA gene of bacterial isolates was amplified by PCR using universal primers 27F (5′ AGAGGTTTGATCCTGGCTCAG 3′) and 1492R (5′ GGTTACCTTGTTACGACTT 3′), and seven housekeeping genes (*glpF, ilvD, ptA, purH, pycA, rpoD*, and *tpiA*) were amplified using primers described by Le et al. (2019). PCR amplifications included a 20 μl mixture containing 1 μl of genomic DNA of the strain EM-1 as a template, 0.5 μl of each primer (0.5 μM), 10 μl of polymerase buffer with dNTPs and MgCl_2_, 0.5 μl of Taq DNA polymerase (TransGen Biotech, China), and distilled water. PCR amplification was performed as follows: one cycle of 5 min at 95°C; followed by 40 cycles of 30 s at 95°C, 30 s at 50°C, and 1.5 min at 72°C; then followed by one cycle of 10 min at 72°C. PCR products were sent to Ruibiotech for Sanger sequencing (Qingdao, China). The 16S rRNA gene sequence was aligned in the EzBioCloud database (https://www.ezbiocloud.net/) (Yoon et al., 2017). The multi-locus sequence analysis (MLSA) (www.pubmlst.org/bsubtilis) was used to analyze housekeeping genes. The nucleotide sequences were concatenated to construct a phylogenetic tree. The phylogenetic tree was constructed using MEGA X software by using the maximum-likelihood method and general time reversible model (Nei and Kumar, 2002; Kumar et al., 2018).

### Efficacy of the isolate EM-1 under greenhouse conditions

Tobacco seedlings with five leaves were divided into two treatment groups (I and II) with three biological replicates, and each replicate contained at least 20 seedlings: (I) irrigating EM-1 suspensions (OD_600_ = 0.6) and then inoculating *Rs* suspensions on the 2nd day; (II) control, irrigating *Rs* suspensions (OD_600_ = 0.7). The bacterial suspensions were prepared, as described previously (Section Screening of candidate bio-control *Bacillus* isolates). The bacterial suspensions of EM-1 and *Rs* were then centrifuged at 6,000 rpm for 5 min and diluted in water to OD_600_ of 0.6 and 0.7, respectively. Each inoculation treatment was 10 ml per plant. Plants were cultivated in a greenhouse, as described before (Section Microbial and plant resources) and watered every 3 days to maintain soil moisture. The disease severity of the tobacco plants was scored on the 5th day of *Rs* inoculation, and tobacco samples were collected for further use. The disease severity investigation and disease index calculation were carried out according to (Zhang et al., 2017).

### Quantification of *R. solanacearum*

Total DNA of root samples were extracted using the FastDNA^®^ Spin Kit for Soil (MP Biomedicals, USA). The specific primer Rsol_*fliC* (F 5′ GAACGCCAACGGTGCGAACT 3′ and R 5′ GGCGGCCTTCAGGGAGGTC 3′) that targets the *fliC* gene encoding the flagellum subunit was used to quantify *Rs* densities. Real-time PCR experiments were performed by using the SYBR^®^ Premix Ex TaqTM (Takara Bio Inc, Japan) and analyzed using the 7500 Real-Time PCR System (Applied Biosystems, USA). Standard curves were generated using 10-fold serial dilutions of a plasmid containing a fragment copy of *Rs fliC*. qPCR amplifications for standard and DNA samples included a 20 μl mixture containing 2 μl of templates, 10 μl of the SYBR Green Premix Ex Taq (2 ×), 0.4 μl of ROX Reference Dye II, 0.4 μl of each primer, and distilled water. PCR amplification was programmed with one cycle of denaturation at 95°C for 30 s and 40 cycles of 95°C for 5 s and 60°C for 34 s. Fluorescence was monitored in each PCR cycle during the annealing and extension phases at 60°C (Zheng et al., 2021).

### Determination of the defense-related enzymes in leaves

The crude enzyme extracts of polyphenol oxidase (PPO), superoxide dismutase (SOD), and catalase (CAT) were prepared as follows: Tobacco leaves (0.1 g) were dipped in liquid nitrogen in a pre-cooled mortar, homogenized in 1 ml of ice-cold 50 mM potassium phosphate buffer (pH 6.8), and centrifuged at 12,000 rpm at 4°C for 10 min. The supernatant was collected as the enzyme extract of SOD and CAT. The preparation of crude PPO was the same, except for sodium borate buffer (pH 8.8). The enzyme activity determination was performed according to Ju et al. (2014), Li et al. (2014), and Wang et al. (2019). Each treatment consisted of three replicates.

### Antibacterial activity of a cell-free culture filtrates (CFs) from strain EM-1

CFs obtained from the strain EM-1 were prepared, as described by Ezrari et al. (2021). In brief, the strain EM-1 was cultured in LB liquid medium, at 180 rpm for 72 h at 28°C, and then the liquid cultures were centrifuged at 8,000 g for 10 min. The supernatants were collected and filtered using 0.22-μm filter membranes and then dried into powder by freeze drying (the temperature and pressure of the vacuum oven were −80°C and 1.0 Pa, respectively). The inhibition zone method was used to evaluate the antibacterial activity of the supernatants, as described by Zhu and Zhang (2020). The powder was diluted with methanol into 50 mg/ml. *Rs* was inoculated into NA medium to prepare a plate, as described before. After that, three holes were punched in each of the plate. Then, 60 μl of CFs and methanol as a control were injected into the holes, and the diameter of the inhibition zone around the hole was measured to determine antibacterial activity after incubation at 28°C for 48 h.

### Extraction of extracellular compounds from strain EM-1

#### Extraction of the secondary metabolites

To prepare the samples for antagonistic activity experiments, cell-free filtrates (CFs, 50 ml, 100 mg/ml) of the strain EM-1 were passed through an Amberlite XAD-16 column (100 g, 4 × 70 cm), as described by Yuan et al. (2012a) with some modifications. In brief, the active compounds and impurities were bound to the column matrix. To remove the impurities, first, the column was washed with 500 ml of 30% methanol, and then elution of active compounds was performed using 500 ml of 100% methanol. The eluates were dried in a rotary evaporator (<40°C). The secondary metabolites were dissolved in methanol to prepare the solution with a concentration of 50 mg/ml.

For the preparation of polyketides, the CFs were extracted with ethyl acetate (Mondol et al., 2011) after EM-1 was incubated in LB medium at 37°C with shaking at 200 rpm for 16 h. The CFs of EM-1 were mixed with ethyl acetate (EtOAc, 1:1 volume ratio) and extracted two times. The cumulative EtOAc layer was dried in a rotary evaporator (<40°C), and the extract was dissolved in methanol to prepare the solution with a concentration of 30 mg/ml. The iturin A standard (Sigma, USA) was purchased and dissolved in methanol to prepare the solution with concentrations of 10, 5, and 1 mg/ml. The activity of the crude extract against *Rs* was evaluated by using the well diffusion method with the same conditions previously used.

#### Extraction of crude protein

Crude protein extracts were precipitated using the ammonium sulfate precipitation method with slight modifications (Wu et al., 2020). The strain EM-1 was grown in 200 ml liquid LB medium and incubated at 180 rpm, 28°C for 72 h. Supernatants were collected by centrifugation at 8,000 g, 4°C for 20 min. The soluble proteins in the supernatants were precipitated by slowly adding two volumes of 100% saturated ammonium sulfate solution, and the resulting protein-containing suspensions were then centrifuged at 12,000 rpm for 30 min. The precipitated proteins were suspended in 20 mM Tris–HCl, pH 7.4, and dialyzed against the same buffer overnight to remove residual ammonium sulfate. The complete removal of ammonium sulfate was confirmed by continuous titration of the dialysate with barium chloride solution (0.5 M) until no white precipitation was observed. The resulting protein-containing solutions were dried into powder by freeze drying (the conditions of freeze drying were as described in Section Antibacterial activity of a cell-free culture filtrates (CFs) from strain EM-1). The crude protein extracts were dissolved in methanol to prepare the solution with a concentration of 50 mg/ml. The activity of the crude protein extracts against *Rs* was evaluated by using the well diffusion method using the same conditions previously used.

### Identification of the active substances

#### Identification of the secondary metabolites by LC-MS/MS

LC-MS/MS was carried out by coupling an Agilent 1290 LC System to a LTQ-Orbitrap XL Mass Spectrometer (Thermo Fisher Scientific, USA). Chromatographic separation was performed on a SunFire column (C18, 250 × 2.6 mm, Waters, USA). The mobile phase was 60% A [0.1% (v/v) aqueous acetic acid] and 40% B [acetonitrile] with 0.6 ml/min flow rate, 30°C column temperature, and 10 μl injection volume. The general mass spectrometric parameters were as follows: the spray voltage, capillary voltage, and tube lens voltage were 4.0 kV, 16 V, and 35 V, respectively. The capillary temperature was 300°C with a sheath gas flow rate of 40 L/min and an auxiliary gas flow rate of 10 L/min. External calibration of mass spectra routinely produced a mass accuracy of better than 3 ppm. Full mass spectra were acquired in the positive ionization mode at a resolution of 30,000 with 100–1,500 Da mass range, followed by a data-dependent scan in the collision-induced dissociation (CID) mode. Data acquisition and analysis were performed using Xcalibur software version 4.1 (Thermo Fisher Scientific, USA).

#### Identification of the volatile organic compounds (VOCs) by GC-MS/MS

VOCs were extracted by headspace solid-phase microextraction (HS-SPME). Airtight vials were thoroughly cleaned, sterilized, and baked at 120°C for 20 h before use. NA medium was poured into the airtight vials to obtain slants. The suspensions of EM-1 were prepared as described earlier (2.3). A measure of 100 μl of suspensions were transferred to an NA slant in an airtight vial, which was sealed tightly with polytetrafluoroethylene (PTFE) septum, cultured at 28°C for 48 h in dark. The culture medium that was subjected to identical conditions was set as the control. For the sampling of volatile compounds, the solid-phase microextraction (SPME) fiber was inserted into the headspace of the airtight vial containing EM-1 at room temperature for 20 min. When the sampling was completed, the SPME fiber was removed from the sample vial and immediately inserted into the GC injector at 250°C for 5 min with a split ratio of 10:1. The used SPME fiber was conditioned at 250°C for 5 min prior before the next sampling.

GC-MS/MS (an Agilent 7890B GC system coupled to a 7000C GC/MS Triple Quad Mass Detector) (Agilent Technologies, USA) was used to identify VOCs. Chromatographic separation was performed on a 30-m DB*-*5MS capillary column (Agilent 122-5532UI, USA), the temperature of the injection ports was 250°C, and the flow rate was 1.5 ml/min. The temperature program started in the isocratic mode at 45°C for 2 min, followed by temperature ramping of 5°C/min to a final temperature of 280°C, which was then held constantly for an additional 2 min. The MS operating parameters were as follows: electron energy 70 eV, ion source temperature 280°C, quadrupole temperature 180°C, and m/z scanned area 33–500. The mass spectra data for the VOCs were analyzed using the data in the NIST/EPA/NIH Mass Spectrum Library.

### Fumigation effect of synthetic VOCs identified through GC-MS analysis

The 2-heptanone standard (98% pure) and 6-methyl-2-heptanone standard (98% pure) were purchased from Aladdin (China), while the 5-methyl-2-heptanone standard (98% pure) was purchased from AndHider (China). These chemical standards were used for the fumigation test with a modified sealed plate method (Tahir et al., 2017). The suspensions of *Rs* (5 μl) were point-inoculated at the center of NB plates, while the chemical standards (20 μl, 98% pure) were taken on a Petri dish cover, sealed with parafilm, and incubated at 28°C. Sterilized water (20 μl) and EM-1 suspensions were taken on a Petri dish cover as negative control and positive control, respectively. The medium containing *Rs* was cut down after 24 h and added into the sterilized water and shaken for 5 min. Cell density was measured at OD_600_ to evaluate T of the VOCs against *Rs*.

### Genomic analysis of strain EM-1

Genomic DNA was extracted using the SDS method (Lim et al., 2016). The harvested DNA was detected by agarose gel electrophoresis and quantified by a Qubit^®^ 2.0 Fluorometer (Thermo Scientific). Libraries for single-molecule real-time (SMRT) sequencing were constructed with an insert size of 10 kb using the SMRTbell TM Template Kit, version 1.0. A total of 1 μg DNA per sample was used as input material for the DNA sample preparations. Sequencing libraries were generated using a NEBNext^®^ UltraTM DNA Library Prep Kit for Illumina (NEB, USA) following manufacturer's recommendations, and index codes were added to attribute sequences to each sample. These libraries were sequenced on the PacBio Sequel platform (Novogene, China), and PacBio reads were assembled using SMRT Link software v5.0.1. The coding genes, repetitive sequences, and noncoding RNA were predicted using GeneMarkS (http://topaz.gatech.edu/), and the Cluster of Orthologous Groups (COG) database (http://www.ncbi.nlm.nih.gov/COG) was used to annotated the functional information of ORFs by DIAMOND software. The complete genome sequence of the strain EM-1 was accessible from the NCBI with GenBank accession number CP095842.

### Statistical analysis

All statistical analyses were carried out using SPSS 21.0 software. We used the *t*-test, one-way analysis of variance (ANOVA), and Tukey's multiple comparison test to determine the significant differences (*p* < 0.05) between treatment groups. All experiments were performed with at least three independent replicates, unless otherwise stated.

## Results

### *Bacillus* strain isolation

**Figure 1:**
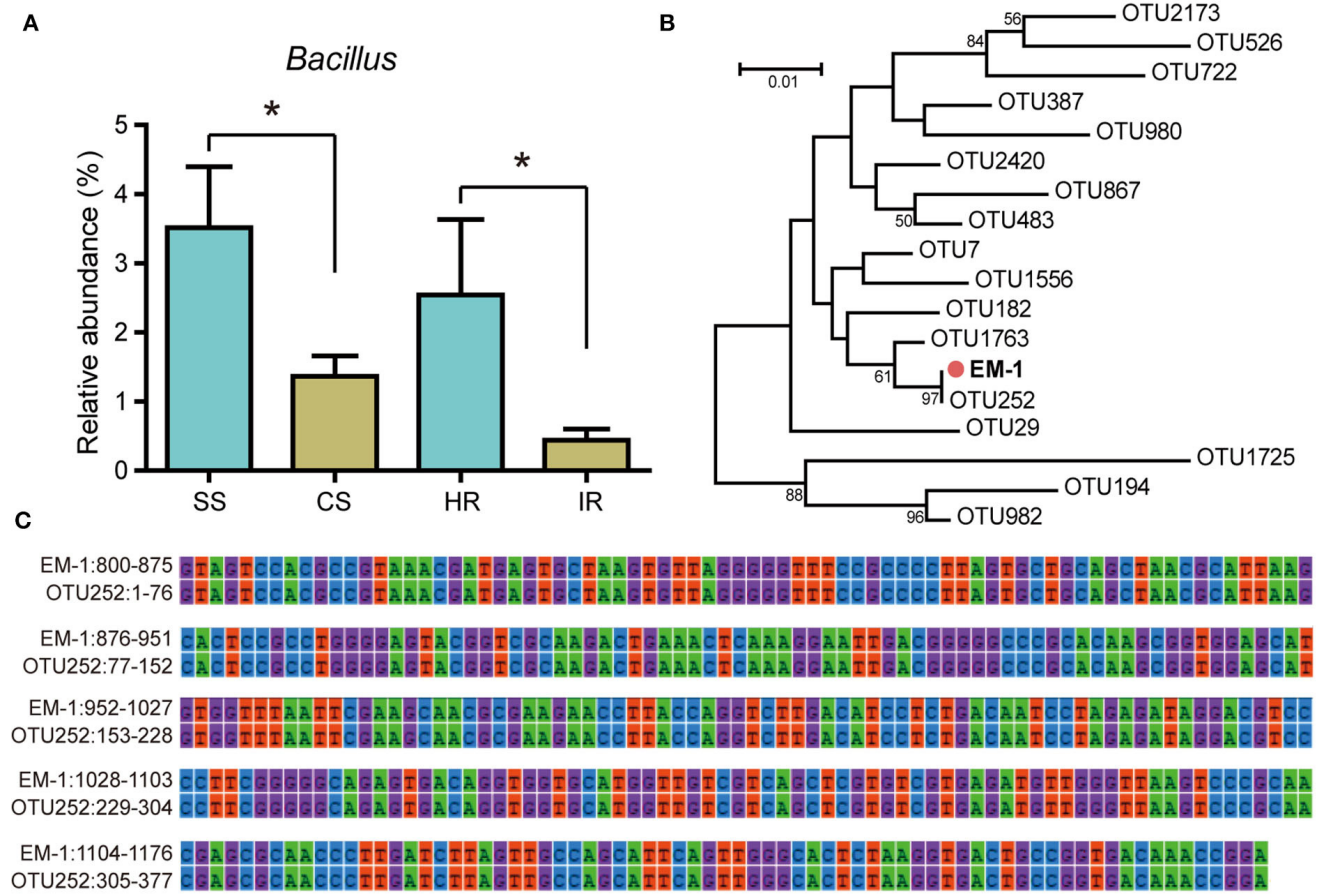
Relative abundance of *Bacillus* OTUs and their phylogenetic relationships with strain EM-1. **(A)** Relative abundance of *Bacillus* OTUs in different samples. SS, suppressive rhizosphere soil; CS, conducive rhizosphere soil; HR, root of healthy tobacco; IR, root of infected tobacco. **(B)** Neighbor-joining phylogenetic tree constructed with the 16S rRNA gene sequences of all *Bacillus* OTUs obtained from high-throughput amplicon sequencing and the strain EM-1. **(C)** 16S rRNA gene alignment between OTU252 and strain EM-1. High-throughput amplicon data used in this figure were derived from our previous study (Zheng et al., 2021).

The relative abundance of genus *Bacillus* was significantly higher in disease-suppressive soil and healthy root samples than that in disease-conducive soil and infected roots (Figure 1A), implying *Bacillus* species might play a critical role in suppressing tobacco bacterial wilt. In this study, we obtained 43 *Bacillus* strains from healthy tobacco rhizosphere soil and root samples. Among them, four isolates showed antagonistic activity against *Rs* (Supplementary Table 1). The strain EM-1 displayed the largest bacteriostatic circle and was selected for further evaluation. To determine whether the strain EM-1 represented indigenous species of bacterial wilt-suppressive soil, phylogenetic relationships between strain EM-1 and *Bacillus* OTUs from high-throughput amplicon data were analyzed. The result indicated that the strain EM-1 clustered together with OTU252 (Figure 1B) and showed 100% 16S rRNA gene similarity to this OTU (Figure 1C), suggesting EM-1 can be considered a resident bacterium in the tobacco rhizosphere.

### Identification of the potential biocontrol strain EM-1

**Figure 2:**
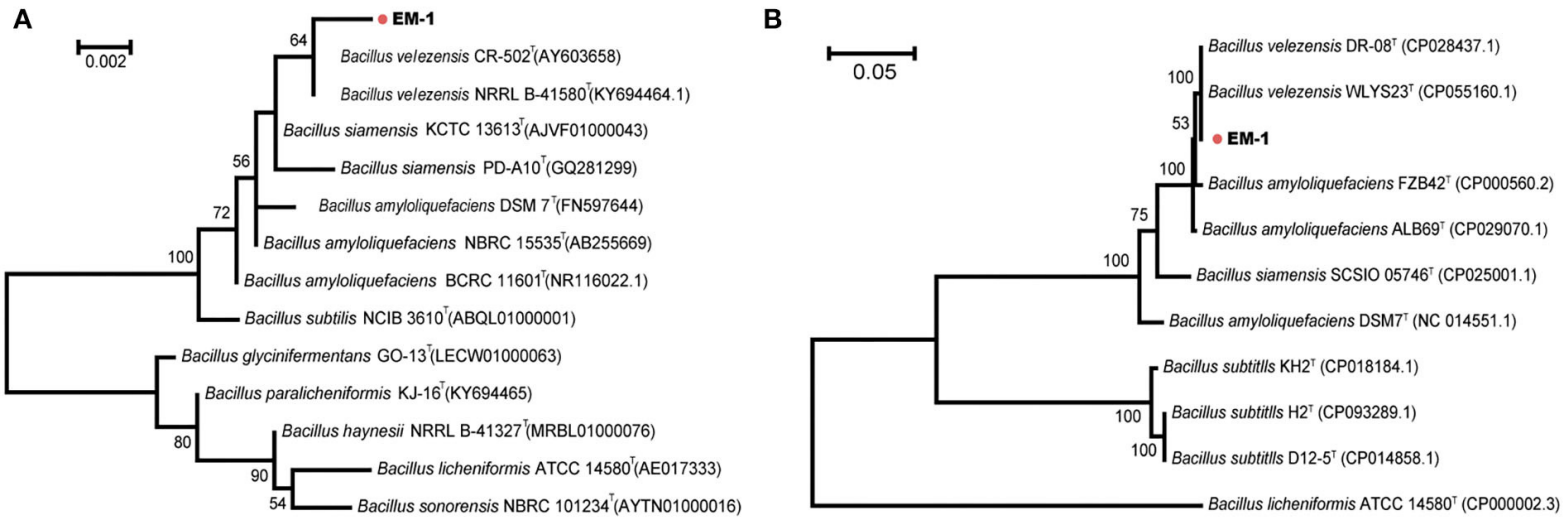
Phylogenetic analysis of strain EM-1. **(A)** Maximum-likelihood phylogenetic tree constructed with 16S rRNA gene sequences. **(B)** Maximum-likelihood phylogenetic tree constructed with the concatenated MLST DNA sequences of the seven housekeeping genes.

Bacterial identification to the species level was frequently performed using 16S rRNA gene sequencing. Sequence alignment of 16S rRNA gene (accession number: OK090956) indicated that the strain EM-1 exhibited 99.79% sequence similarity to *Bacillus velezensis* CR-502^T^ (accession number: AY603658). For the MLSA tree, we used the nucleotide sequences of the seven genus *Bacillus*-specific MLST genes: *glpF, ilvD, ptA, purH, pycA, rpoD*, and *tpiA*. The final sequence length was 2,871 bp. The phylogenetic analyses based on both the 16S rRNA gene and housekeeping gene sequences revealed that the strain EM-1 was closely related to members of *B. velezensis* species (Figure 2).

### Antagonistic ability of the strain EM-1 *in vitro*

**Figure 3:**
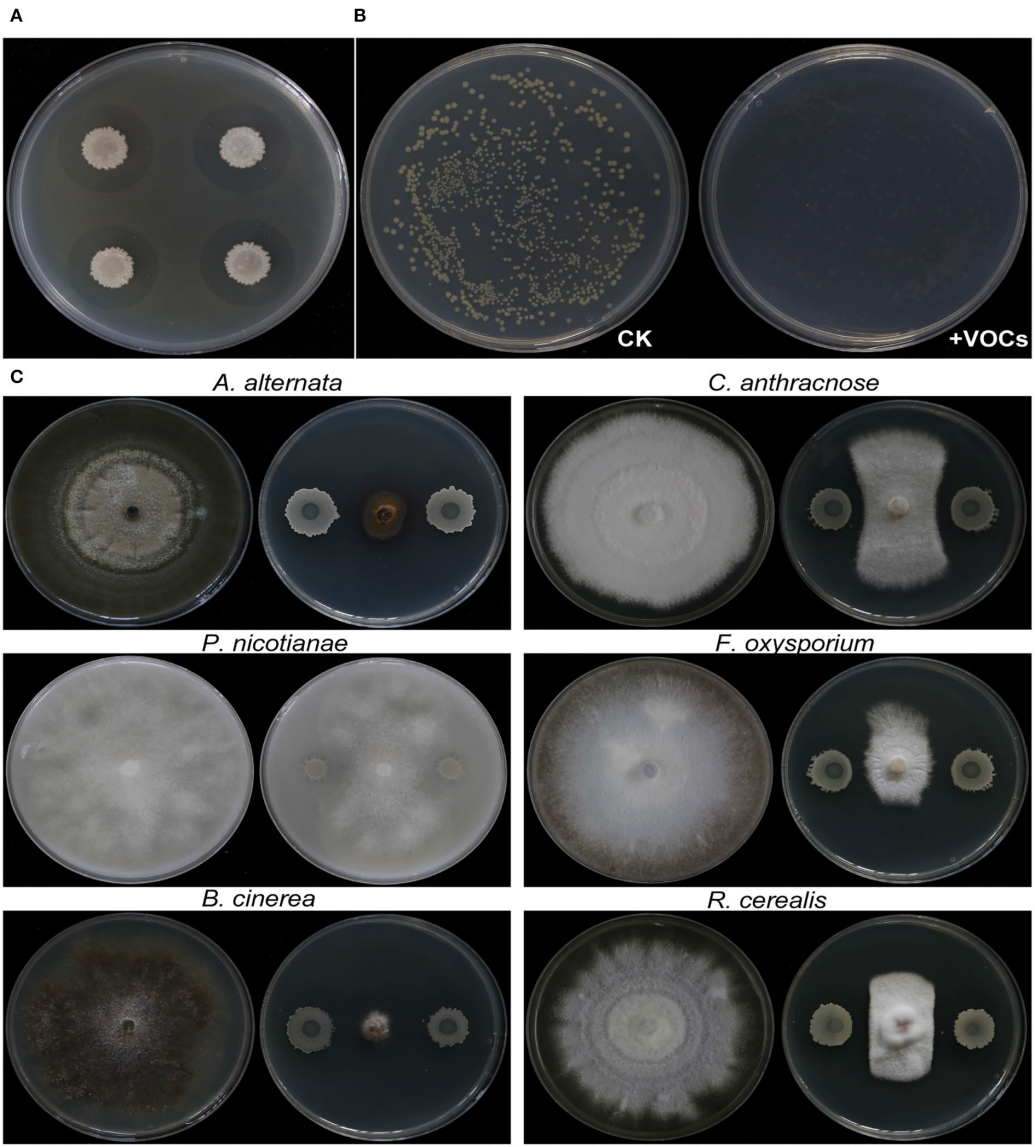
Antagonistic activity of strain EM-1 against *R. solanacearum* and antifungal activity against different pathogenic fungi. **(A)** Antagonistic activity of strain EM-1 against *R. solanacearum*. **(B)** Effects of VOCs produced by strain EM-1 against *R. solanacearum*. **(C)** Antifungal activity of strain EM-1 against different pathogenic fungi.

The growth of *Rs* was inhibited not only by EM-1 (Figure 3A), but also by VOCs produced by EM-1 (Figure 3B), indicating a strong fumigation effect of EM-1 on *Rs*. EM-1 also showed a broad-spectrum antagonistic activity against a variety of phytopathogens, including *A. alternata, P. nicotianae, B. cinerea, C. anthracnose, F. oxysporum*, and *R. cerealis* (Figure 3C). Among them, the strain EM-1 showed the strongest antagonistic effect on *B. cinerea*, followed by *A. alternata*, and the inhibition rates were 89.5 and 79.4%, respectively (Supplementary Figure 1).

### Biocontrol efficacy of the strain EM-1 against tobacco bacterial wilt in greenhouse-grown plants

**Figure 4:**
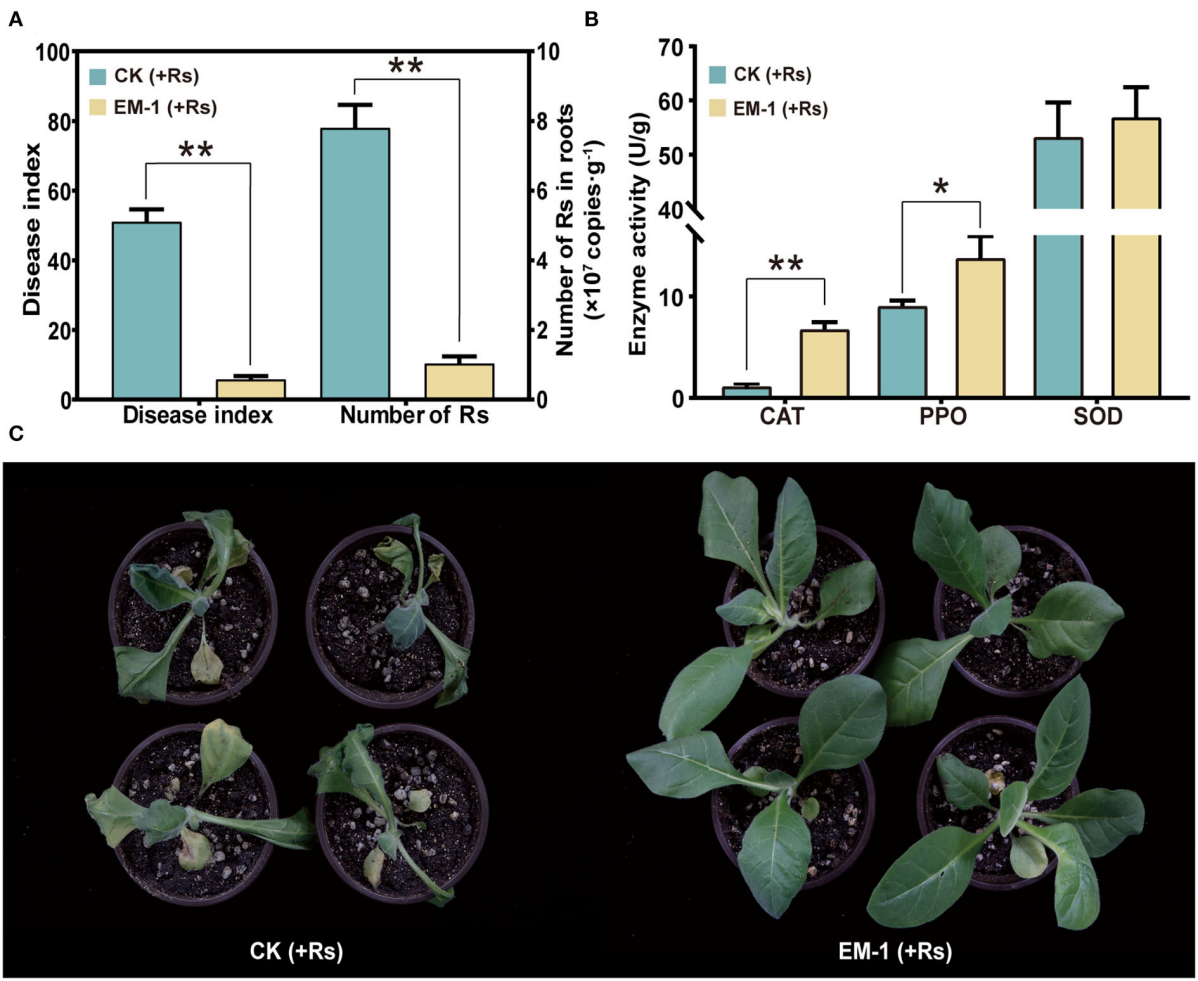
Defense-related enzyme activity and biocontrol effect of strain EM-1 on *R. solanacearum*. **(A)** Disease index of strain EM-1 against tobacco bacterial wilt and the absolute abundance of *R. solanacearum* in root samples. **(B)** Defense-related enzyme activity in leaves. Asterisks indicate a significant difference between the control and EM-1-treated plants (*t*-test, *p < 0.05, **p < 0.001). **(C)** Disease development symptoms in leaves observed at 5 days post-inoculation with *R. solanacearum* in control and EM-1-treated tobacco.

Based on the qPCR result, the abundance of *Rs* in roots inoculated with the strain EM-1 was 1.03 × 10^7^ copies/g, while that in the control was 7.8 × 10^7^ copies/g, which was up to 8-fold higher than that in the strain EM-1-treated group (Figure 4A). This result suggested that the strain EM-1 significantly reduces the colonization of *Rs* in tobacco roots.

After 3 days of *Rs* inoculation, the symptoms of wilt gradually appeared on the control plants. On the 5th day, all the control plants were infected and accompanied by varying degrees of wilting (Figure 4C), reaching a disease index of 51.03. By contrast, a small amount of tobacco plants treated with the strain EM-1 showed disease symptoms, with the disease index of 5.8 (Figure 4A). These results indicated that the strain EM-1 has potential to control tobacco wilt disease.

### Induction of resistance-related enzymes in tobacco leaves

To analyze the impact of inoculating EM-1 on tobacco resistance to the *Rs* pathogen, SOD, CAT, and PPO activities in the leaf samples were measured. Compared with the control group, SOD activity had no significant difference, while CAT and PPO activities of the EM-1 treatment were significantly increased (Figure 4B). These results indicate that the application of the strain EM-1 could induce the activity of defense-related enzymes in tobacco seedlings.

### Inhibitory effect of extracellular compounds of EM-1

**Figure 5:**
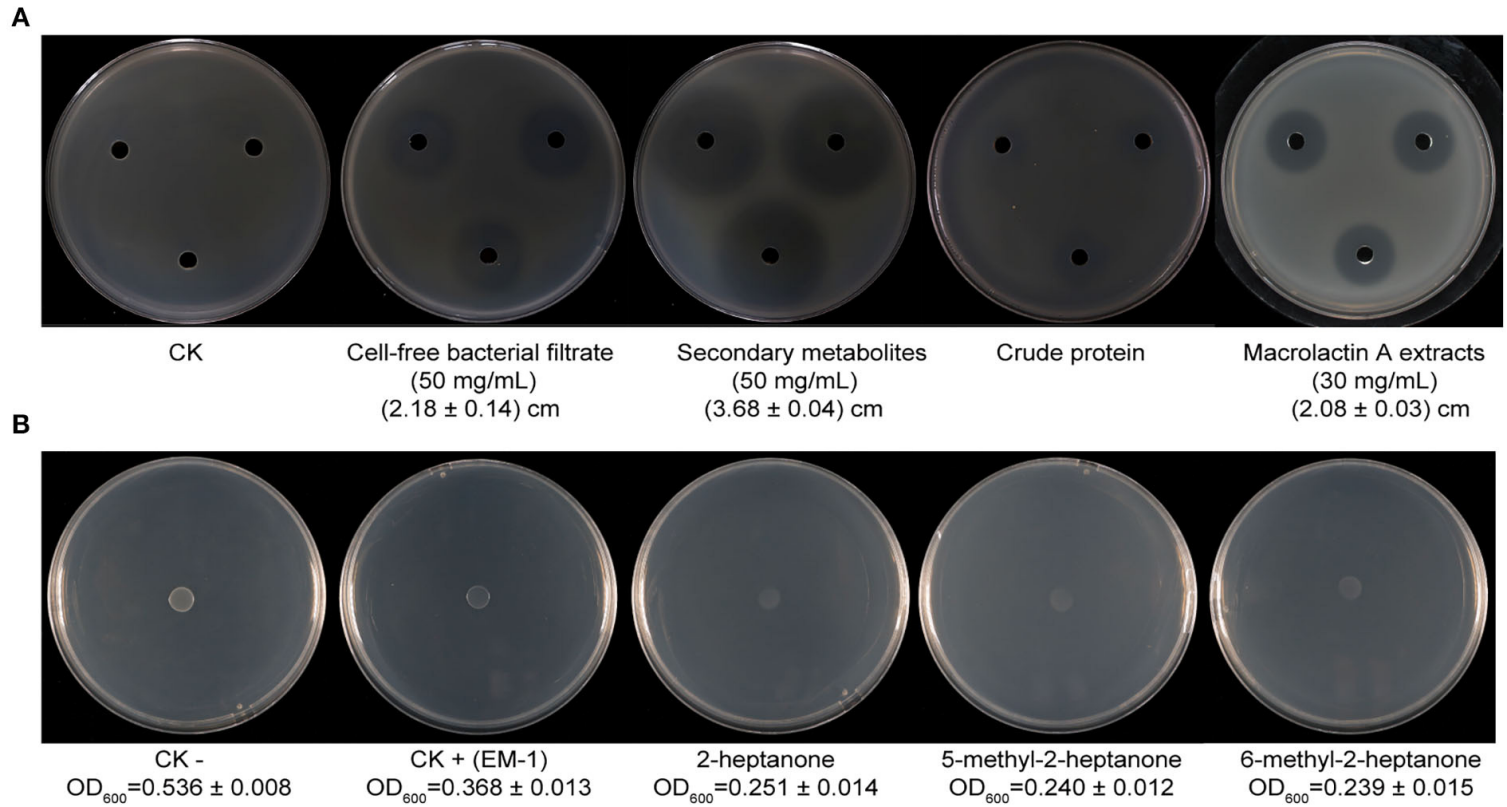
Inhibition of bacterial metabolites of strain EM-1 and VOC standards against *R. solanacearum in vitro*. **(A)** Inhibition of bacterial metabolites of strain EM-1 against *R. solanacearum*. **(B)** Inhibition of VOC standards against *R. solanacearum*.

A preliminary experiment indicated that the cell-free supernatant of EM-1 can effectively inhibit the growth of *Rs*. The results showed that the inhibition zone diameter produced by the cell-free supernatant (50 mg/ml) is 2.18 ± 0.14 cm (Figure 5A), which indicated that the biocontrol ability of the strain EM-1 could be partially attributed to the production of extracellular bioactive substances.

The secondary metabolites and proteins produced by EM-1 were further extracted, and their inhibition ability against *Rs* was assayed. The results showed that the secondary metabolites (50 mg/ml) have a significant inhibitory effect on *Rs* with the inhibition zone diameter of 3.68 ± 0.04 cm, which is larger than those produced by the simple cell-free culture filtrates (50 mg/ml, 2.18 ± 0.14 cm). However, there is only weak inhibition zone produced by crude protein extracts (Figure 5A). Thus, it can be concluded that the secondary metabolites may play a major role on the antagonistic ability of EM-1 against *Rs*.

### Identification and suppression effect of the secondary metabolites produced by EM-1

The chromatograms of LC-MS analyses are shown in Supplementary Figure 2, and the prominent masses from the LC-MS spectra are listed in Table 1. The spectra of compounds revealed that compound A with a molecular mass (m/z) of 1,043.56 Da [M + H]^+^ might be iturin A (C14; Supplementary Figure 2B), compounds C–E with an m/z of 1,057.57 Da [M + H]^+^ might be iturin A (C15; Supplementary Figures 2D–F), and compound F with an m/z of 1,071.59 Da[M + H]^+^ might be iturin A (C16; Supplementary Figure 2G). The three substances with m/z values of 1,043.56, 1,057.57, and 1,071.59 Da exhibited a 14-Da (–CH2) difference in molecular weights, the same difference previously reported for iturin A (Yuan et al., 2012b). Compounds C–E with the same molecular mass might be isomers of iturin A (C15); two other compounds B and G with molecular masses of 687.3 Da and 425.23 Da [M + Na]^+^ were found to be macrolactin W (Supplementary Figure 2C) and macrolactin A (Supplementary Figure 2H), respectively. The polyketides were extracted and identified as macrolactin A by the UV absorbance spectrum and LC-MS/MS (Supplementary Figure 3). The macrolactin A extracts (30 mg/ml) showed an inhibitory effect on *Rs* with an inhibition zone diameter of 2.08 ± 0.03 cm (Figure 5A). In addition, the iturin A standard did not show an inhibitory effect on *Rs* at the tested concentration (10, 5, and 1 mg/ml) (Supplementary Figure 4), indicating that macrolactins might be the main active substances against *Rs*.

**Table 1:** Secondary active metabolite compounds of *B. velezensis* EM-1 detected by LC-MS/MS.

**Number**	**Retention time (min)**	**Ions**	**M/Z**	**Molecular weights**	**Compounds**	**References**
A	13.54	[M+Na]^+^	1,065.54	1,042.5	Iturin A (C14)	Yuan et al. (2012b)
		[M+H]^+^	1,043.56			
		[M+2H]^2+^	522.28			
B	14.53	[M+Na]^+^	687.3	664.2	Macrolactin W	Mondol et al. (2011)
		[M+NH_4_]^+^	682.35			
		[M-H_2_O+H]^+^	647.31			
C	19.00	[M+Na]^+^	1,079.55	1,056.5	Iturin A (C15)	Yuan et al. (2012b)
		[M+H]^+^	1,057.57			
		[M+2H]^2+^	529.29			
D	20.15	[M+Na]^+^	1,079.55	1,056.5	Iturin A (C15)	Yuan et al. (2012b)
		[M+H]^+^	1,057.57			
		[M+2H]^2+^	529.29			
E	22.11	[M+Na]^+^	1,079.55	1,056.5	Iturin A (C15)	Yuan et al. (2012b)
		[M+H]^+^	1,057.57			
		[M+2H]^2+^	529.29			
F	34.26	[M+Na]^+^	1,093.57	1,070.5	Iturin A (C16)	Yuan et al. (2012b)
		[M+H]^+^	1,071.59			
		[M+K+H]^2+^	555.27			
		[M+2H]^2+^	536.3			
G	39.12	[2M+Na]^+^	827.47	402.2	Macrolactin A	Romero-Tabarez et al. (2006)
		[M+Na]^+^	425.23			
		[M+NH_4_]^+^	420.28			
		[M-2H_2_O+H]^+^	367.23			
		[M-3H_2_O+H]^+^	349.22			

### Identification and fumigation effect of VOCs produced by EM-1

A total of 23 different substances were detected as VOCs of EM-1 by GC-MS analysis (Table 2). Among them, 2-heptanone (27.94%), 6-methyl-2-heptanone (12.66%), and 5-methyl-2-heptanone (10.82%) were the main components, representing approximately 51% of total VOCs. The standard chemicals of the three components were purchased and tested for their fumigation effect on *Rs*. The VOC standards exhibited significant antibacterial activity against *Rs*, where the cell densities of *Rs* exposed to 2-heptanone, 6-methyl-2-heptanone, or 5-methyl-2-heptanone were OD_600_ = 0.251 ± 0.014, OD_600_ = 0.239 ± 0.015, and OD_600_ = 0.240 ± 0.012, respectively, while cell densities of the negative and positive controls were OD_600_ = 0.536 ± 0.008 and OD_600_ = 0.368 ± 0.013, respectively (Supplementary Figure 5).

**Table 2:** VOCs of *B. velezensis* EM-1 detected by GC-MS/MS.

**Retention time (min)**	**Peak area (Ab × s)**	**Compounds**	**Structure**	**CAS number**
2.083	6,089,962	2-Pentanone	C_5_H_10_O	107-87-9
2.878	4,131,405	2-Pentanone, 3-methyl-	C_6_H_12_O	565-61-7
3.47	938,228	2-Hexanone	C_6_H_12_O	591-78-6
4.932	5,091,560	2-Hexanone, 5-methyl-	C_7_H_14_O	110-12-3
5.772	28,524,854	2-Heptanone	C_7_H_14_O	110-43-0
7.572	12,929,262	2-Heptanone, 6-methyl-	C_8_H_16_O	928-68-7
7.83	11,046,406	2-Heptanone, 5-methyl-	C_8_H_16_O	18217-12-4
7.93	1,171,730	2-Heptanol, 6-methyl-	C_8_H_18_O	4730-22-7
8.639	1,370,092	2-Octanone	C_8_H_16_O	111-13-7
10.561	3,806,347	2-Nonanone	C_9_H_18_O	821-55-6
10.952	846,076	2-Nonanol	C_9_H_20_O	628-99-9
11.675	4,268,365	2-Nonanone	C_9_H_18_O	821-55-6
12.016	2,855,316	2-Nonanol	C_9_H_20_O	628-99-9
13.595	1,975,768	2-Decanone	C_10_H_20_O	693-54-9
13.746	4,404,820	2-Decanone	C_10_H_20_O	693-54-9
13.915	1,402,350	2-Decanol	C_10_H_22_O	1120-06-5
14.116	1,339,801	2-Decanol	C_10_H_22_O	1120-06-5
17.502	2,447,264	2-Undecanone	C_11_H_22_O	112-12-9
17.76	1,121,070	2-Undecanol	C_11_H_24_O	1653-30-1
19.222	3,089,641	2-Dodecanone	C_12_H_24_O	6175-49-1
19.46	1,712,747	2-Dodecanol	C_12_H_26_O	10203-28-8
20.171	295,654	2-Dodecanone	C_12_H_24_O	6175-49-1
22.719	1,233,408	2-Tridecanone	C_13_H_26_O	593-08-8

### Genomic analysis of strain EM-1

**Figure 6:**
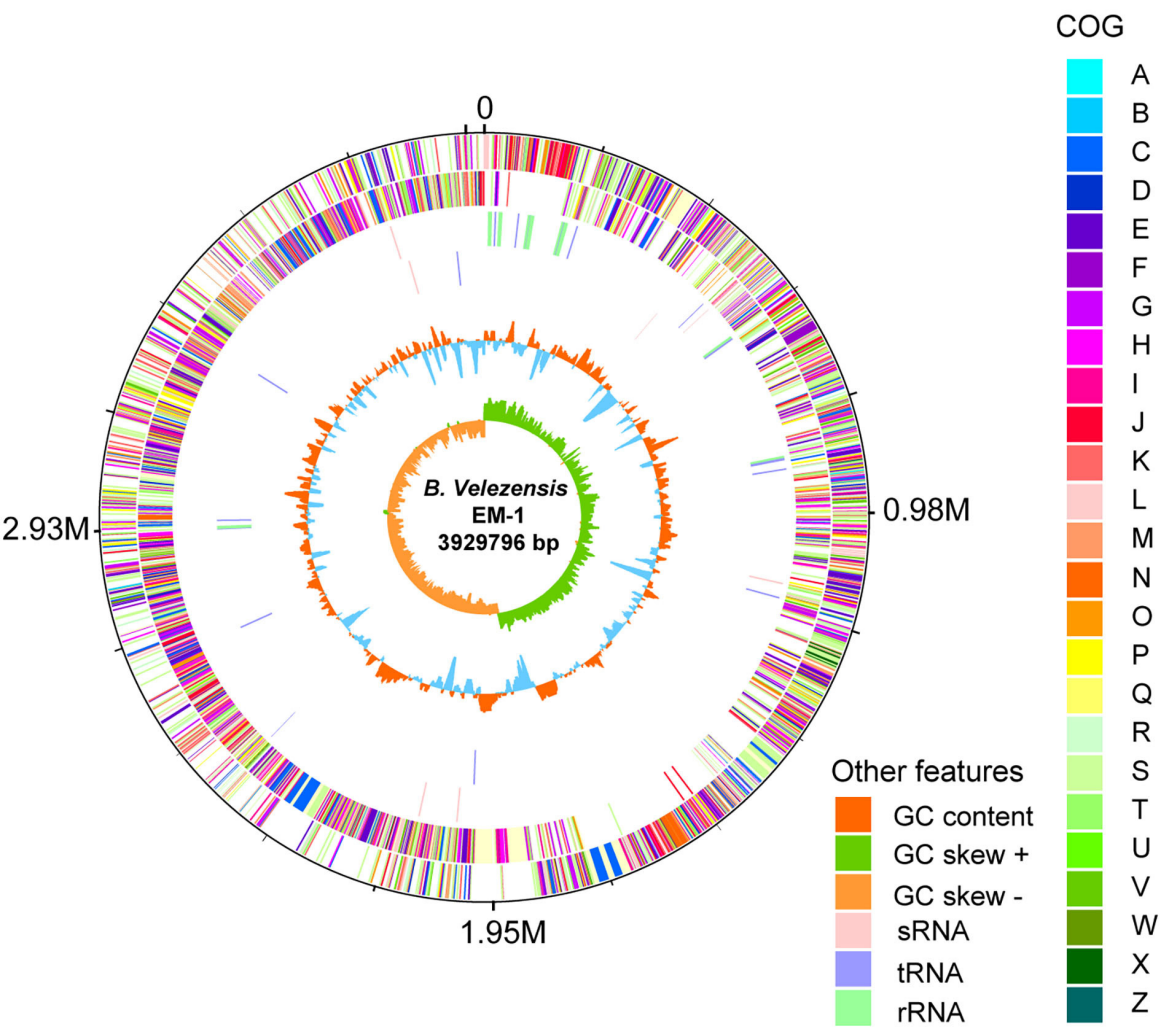
Circular representation of *B. velezensis* EM-1 genome. The outer scale is in mega bases (M). Circle 1 (from outside to inside): the marker of genome size. Circle 2 and 3: CDS with positive and negative chains; different colors represent different functional classifications. Circle 4 and 5: sRNA, tRNA, and rRNA with positive and negative chains. Circle 6: GC content; higher values are redder, and lower are bluer. Circle 7: the GC skew value; the algorithm is (G – C)/(G + C). Generally, when GC skew > 0, the histogram is outward and expressed in green; when GC skew <0, the histogram is inward and expressed in orange.

To investigate the genetic basis of the antimicrobial phenotypes of the strain EM-1, we further analyzed its complete genome (Figure 6). The genome of *B. velezensis* EM-1 consisted of a circular chromosome of 3,929,796 bp in size, with an average GC content of 46.5%. Further analysis predicted that the chromosome contains 4,026 coding DNA sequences (CDSs), 86 tRNAs, 27 rRNAs, and nine sRNAs (Supplementary Table 2).

Based on COG analysis (Tatusov et al., 2003), 2,900 proteins were classified into 25 different functional categories. Among them, 42.97% of proteins were associated with metabolism including “energy production and conversion (5.21%),” “amino acid transport and metabolism (9.08%),” “nucleotide transport and metabolism (2.61%),” “carbohydrate transport and metabolism (7.97%),” “coenzyme transport and metabolism (5.48%),” “lipid transport and metabolism (4.10%),” “inorganic ion transport and metabolism (5.21%),” and “secondary metabolites biosynthesis, transport, and catabolism (3.30%)” (Supplementary Figure 6).

**Figure 7:**
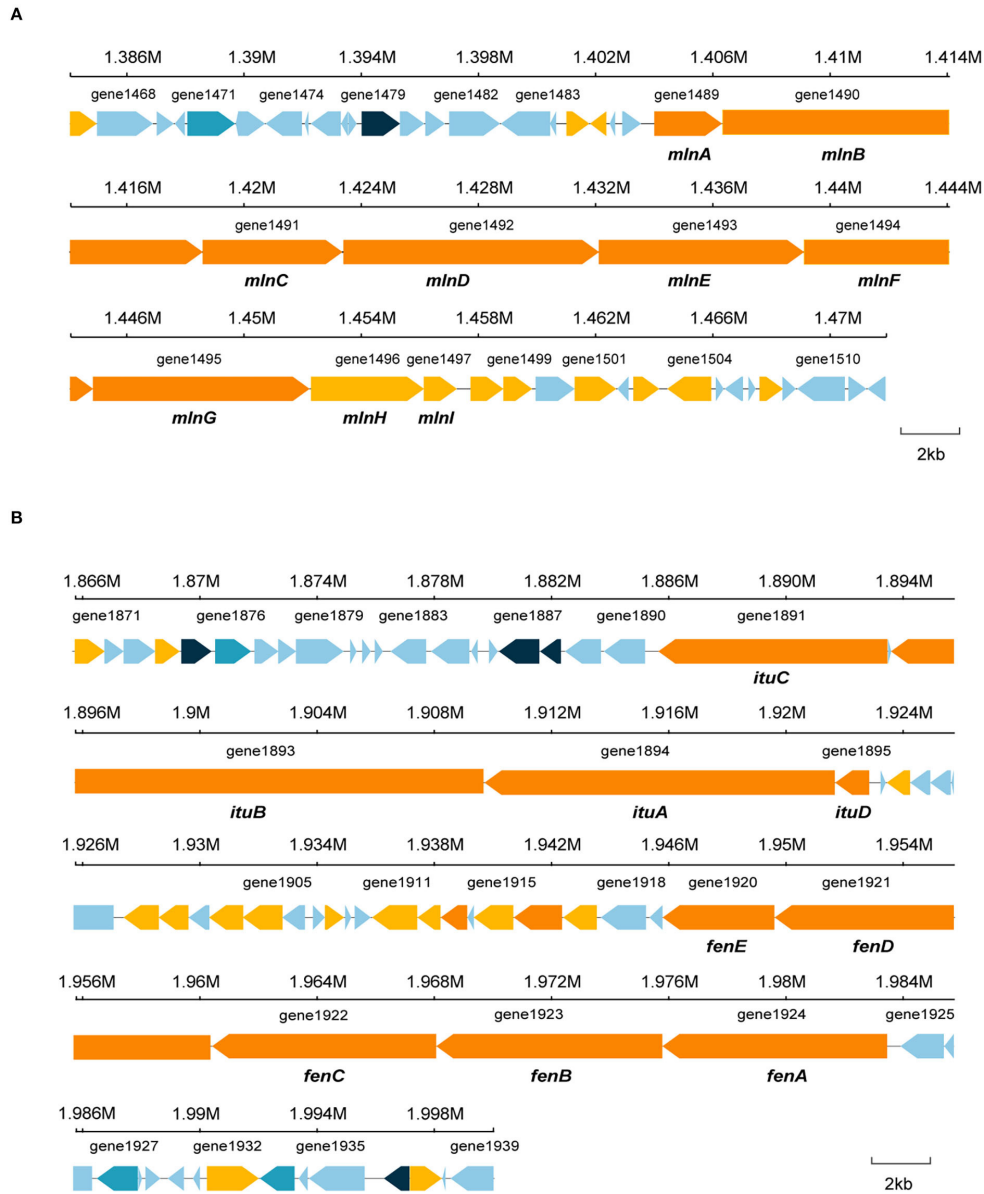
Specific gene structures in cluster 5 and cluster 7. **(A)** Secondary metabolite gene in cluster 5. **(B)** Secondary metabolite gene in cluster 7.

*Bacillus* species has potential to produce secondary metabolites with a wide structural variability that exhibit strong antibacterial and antifungal activity (Sansinenea and Ortiz, 2011). Approximately 18.43% of *B. velezensis* EM-1 genome encodes for genes involved in the production of secondary metabolites with antimicrobial properties. Overall, 12 secondary metabolite biosynthesis gene clusters were identified (Table 3), including surfactin, butirosin, macrolactin, bacillaene, fengycin, difficidin, bacillibactin, bacilysin, and some unknown substances. Cluster 5 displayed high similarity to the macrolactin biosynthetic gene cluster. Macrolactin has been correlated with the gene organization of the *mln* operon, which comprises nine genes *mlnA–I* located in cluster 5 (Figure 7A). Interestingly, the biosynthetic cluster of iturin A was not found using antiSMASH. Based on in-depth analysis of the prediction results, cluster 7 had two core operons: one operon showed high sequence similarity to the fengycin operon of *Bacillus amyloliquefaciens* FZB42, and the other operon showed high sequence similarity to the iturin A operon of *B. subtilis* RB14 (Supplementary Table 3). Iturin A has been correlated with the gene organization of the *itu* operon, which comprises four genes *ituA–D* located in cluster 7 (Figure 7B).

**Table 3:** Secondary metabolic gene clusters of *B. velezensis* EM-1.

**Cluster number**	**Type**	**Size (bp)**	**Most similar known cluster**		**Similarity (%)**
1	NPRS	63,978	Surfactin	NRP:Lipopeptide	82
2	PKS-like	41,245	Butirosin A/butirosin B	Saccharide	7
3	terpene	17,409	–	–	–
4	Lanthipeptide-class-ii	28,889	–	–	–
5	transAT-PKS	87,836	Macrolactin H	Polyketide	100
6	transAT-PKS, T3PKS, NRPS	100,566	Bacillaene	Polyketide+NRP	100
7	NRPS, transAT-PKS, betalactone	134,311	Fengycin	NRP	100
8	terpene	21,884	–	–	–
9	T3PKS	41,101	–	–	–
10	transAT-PKS	93,793	Difficidin	Polyketide+NRP	100
11	NRPS, RiPP-like	51,792	Bacillibactin	NRP	100
12	other	41,419	Bacilysin	Other	100

## Discussion

The microbe–microbe and microbe–root interactions are very complex, which result in different microbial fitness, population dynamics, and functional capacities in the rhizosphere (Banerjee et al., 2018). However, the majority of microbes used as biocontrol agents have been obtained by conventional methods such as isolation, identification, and evaluation. Recently, the core microbiome has been suggested to play a crucial role in an ecosystem; thus, the keystone species can be a promising way to explore effective biocontrol microbes. In our previous study, three *Pseudomonas* strains were identified as the keystone species in suppressive soil and/or healthy plants (Zheng et al., 2021) and showed multiple functions in disease control (Shang et al., 2021). Although the field control effect needs to be further confirmed, these results have indicated that the strategy based on keystone species exploring is effective in finding suitable biocontrol agents. More microbiome-based bioagents will be applied in future. In this study, *B. velezensis* EM-1 associated with suppressive rhizosphere soil microbiome was screened.

Recently, there is increasing research and application of *B. velezensis* in agriculture. Due to its pronounced ability for disease prevention and plant growth promotion, this species has been widely reported for plant disease biocontrol, including potato late blight (Yan et al., 2021), cucumber Fusarium wilt (Luo et al., 2019), lotus rot (Wang et al., 2020), tomato bacterial wilt (Agarwal et al., 2020), rice blast (Jing et al., 2020), and other plant diseases. However, there are few reports on the use of *B. velezensis* to control tobacco bacterial wilt. The current study presents a new *B. velezensis* strain EM-1 with potential for tobacco bacterial wilt biocontrol. EM-1 inoculation can significantly reduce the root colonization of *Rs* and decrease the disease severity of bacterial wilt. Moreover, EM-1 also exhibited inhibitory activity against other fungal and oomycete pathogens. The broad-spectrum antagonistic activity can be beneficial for its biocontrol ability since many pathogens often coexist in the same niche (Chávez-Ramírez et al., 2020).

Currently, the biocontrol mechanism of *B. velezensis* reported mainly include plant growth promotion (PGP), induced system resistance, and antibiotic production (Azabou et al., 2020; Guo et al., 2020; Xu et al., 2021); one antagonist will often exhibit more than one biocontrol mechanism. For instance, strain *B. velezensis* C2 can effectively control *Verticillium* wilt disease not only by antifungal activity but also associating its PGP traits such as siderophore and indole-3-acetic acid production and inorganic phosphate solubilization (Dhouib et al., 2019). As reported previously, the strain EM-1 also showed multiple biocontrol abilities, including significant inhibitory activity on *Rs*, fumigation effect, lipopeptides, and polyketide production and induced resistance of tobacco. These traits can contribute to disease controlling ability directly, indirectly, or synergistically. Here, we pay more attention on the inhibitory substances produced by EM-1.

Previous reports indicated that antimicrobial substances produced by *B. velezensis* mainly consisted of proteins (Guo et al., 2020), lipopeptides, and polyketide antibiotics, which are synthesized by non-ribosomal peptide synthetases (NRPSs) and polyketide synthases (PKSs) (Rabbee et al., 2019; Liu et al., 2020). Our results indicated that secondary metabolites were the main inhibitory substances secreted by EM-1, which showed a significant inhibition effect on *Rs* even at a concentration of 50 mg/ml. They were further identified as iturin A (C14, C15, and C16), macrolactin A, and macrolactin W. To investigate the biosynthetic pathway of iturin A and macrolactin, the whole-genome sequencing of the strain EM-1 was conducted in our study. A total of 12 biosynthetic gene clusters (BGCs) for secondary metabolite synthesis were predicted by antiSMASH. It revealed that cluster 5 in the strain EM-1 encoded a polyketide synthase (PKS), and nine core genes in this cluster were proposed to be responsible for macrolactin biosynthesis. Cluster 7 encoded a hybrid modular polyketide synthase–non-ribosomal peptide synthetase (PKS-NRPS), and four core genes in this cluster were proposed to be responsible for iturin A biosynthesis. Although cluster 7 was predicted to produce “fengycin,” in-depth mining of the prediction results showed that cluster 7 has two core operons, where one has been implicated in regulating fengycin synthesis (Koumoutsi et al., 2004) and the other related to synthesis of iturin A (Tsuge et al., 2001). As outlined previously, the iturin A standard had no inhibitory effect on *Rs* growth, which was consistent with the finding that iturin A was often related to antifungal activity (Arrebola et al., 2010). It has been reported that macrolactin family compounds are effective bacteriostatic antibiotics that inhibit a number of Gram-positive bacterial pathogens (Yuan et al., 2014). The aforementioned results of this study have also demonstrated that macrolactin A extracts could inhibit the growth of *R*s. Polyketides, but not iturins, might be the main active substances in CFs of EM-1 against *Rs*.

Several *B. velezensis* strains have been found to have a fumigation effect on phytopathogens (Lim et al., 2017; Myo et al., 2019; Zhang et al., 2021). The potent active compounds include benzaldehyde, diacetyl, pyrazine (2,5-dimethyl), benzothiazole, phenol (4-chloro-3-methyl), and 2,4-dimethyl-6-tert-butylphenol (Gao et al., 2017; Calvo et al., 2020; Li et al., 2020). However, these reports were mainly concerned with the antifungal activity of VOCs produced by *B. velezensis*. The components against bacterial pathogens are not elucidated yet. The current study identified three compounds (2-heptanone, 6-methyl-2-heptanone, and 5-methyl-2-heptanone) from VOCs of EM-1, which showed a significant fumigation effect on *Rs*. Among them, 6-methyl-2-heptanone was also detected in the VOCs of *B. velezensis* C16 and exhibited antifungal activity against *Alternaria solani* (Zhang et al., 2021). To the best of our knowledge, this is the first report of the three volatile compounds having an inhibitory effect on *Rs*. A comprehensive evaluation of these compounds is definitely worth exploring.

## Conclusion

In summary, the strain *B. velezensis* EM-1 associated with suppressive rhizosphere soil microbes showed high potential for tobacco bacterial wilt control. Antibiotic production, fumigation effect, and induced resistance of tobacco appeared to be involved in its biocontrol mechanisms. Polyketides and VOCs might be the main active substances against *Rs*, and 2-heptanone, 6-methyl-2-heptanone, and 5-methyl-2-heptanone were identified as promising volatile compounds. Overall, these results indicated that EM-1 can act as a biocontrol strain for tobacco bacterial wilt control. Future work should identify the effective polyketides and evaluate the biocontrol effect of EM-1 under field conditions.

## Data availability statement

The data presented in the study are deposited in the https://www.ncbi.nlm.nih.gov/genbank repository, accession number CP095842.

## Author contributions

CZ, CM, and YZ provided the experimental ideas and the design of this study. XS performed the experiment and wrote the manuscript with assistance of XH and JC. YL and YY helped analyze the data and revise the manuscript. JG helped revise the manuscript. All authors contributed to the article and approved the submitted version.

## Funding

This research was supported by Key Science and Technology Projects of China National Tobacco Corporation (110201902003), Science and Technology Project of Guizhou Tobacco Corporation (201809), National Natural Science Foundation of China (32101289), and the Agricultural Science and Technology Project of Southwest Guizhou [110202101057(LS-17)].

## Conflict of interest

Authors XH and JG were employed by Guizhou Tobacco Company, Zunyi. The remaining authors declare that the research was conducted in the absence of any commercial or financial relationships that could be construed as a potential conflict of interest.

## Publisher's note

All claims expressed in this article are solely those of the authors and do not necessarily represent those of their affiliated organizations, or those of the publisher, the editors and the reviewers. Any product that may be evaluated in this article, or claim that may be made by its manufacturer, is not guaranteed or endorsed by the publisher.

## Supplementary material

Supplementary Figure 1 Percentage inhibition of strain EM-1 against different pathogenic fungi. Percentage inhibition, relative to the control, of six plant pathogenic fungi by strain EM-1. All experiments were independently performed in triplicate. Data represent the mean ± standard deviation of three replicates in each treatment group. Different letters indicate a significant difference (*p* < 0.05; Tukey's test) between the treatment and control.

Supplementary Figure 2 Secondary active metabolite compounds detected by LC-MS/MS. **(A)** LC chromatogram of the seven detected antimicrobial compounds. **(B)** Mass spectra of iturin A (C14). **(C)** Mass spectra of macrolactin W. **(D)** Mass spectra of iturin A (C15). **(E)** Mass spectra of iturin A (C15). **(F)** Mass spectra of iturin A (C15). **(G)** Mass spectra of iturin A (C16). **(H)** Mass spectra of macrolactin A. The mass spectra were all detected in the positive ion mode.

Supplementary Figure 3 LC-MS/MS spectrums of macrolactin A. **(A)** LC chromatogram of macrolactin A. **(B)** UV absorption spectra of macrolactin A. **(C)** MS/MS spectrums of macrolactin A.

Supplementary Figure 4 The inhibitory effect of iturin A standard on *Rs*.

Supplementary Figure 5 The cell density of the *Rs* after VOCs treatments.

Supplementary Figure 6 COG functional classification in *B. velezensis* EM-1 coding sequences.

Supplementary Table 1 Details of all *Bacillus* strains isolated from tobacco rhizosphere soil and their inhibition effect against *R. solanacearum*.

Supplementary Table 2 General genome feature of *B. velezensis* EM-1.

Supplementary Table 3 Comparison of core biosynthetic genes of *B. velezensis* EM-1 with known gene clusters in cluster 7.
