# Supplementary material for: Biocontrol potential of Bacillus velezensis EM-1 associated with suppressive rhizosphere soil microbes against tobacco bacterial wilt
Source: Front Microbiol. 2022 Aug 23;13:940156. doi: 10.3389/fmicb.2022.940156 (PMC9445557; doi:10.3389/fmicb.2022.940156)
Supplement: Supplementary Figure 1 — Percentage inhibition of strain EM-1 against different pathogenic fungi. Percentage inhibition, relative to the control, of six plant pathogenic fungi by strain EM-1. All experiments were independently performed in triplicate. Data represent the mean ± standard deviation of three replicates in each treatment group. Different letters indicate a significant difference (p < 0.05; Tukey's test) between the treatment and control. [file Data_Sheet_1.PDF]

Figure S1

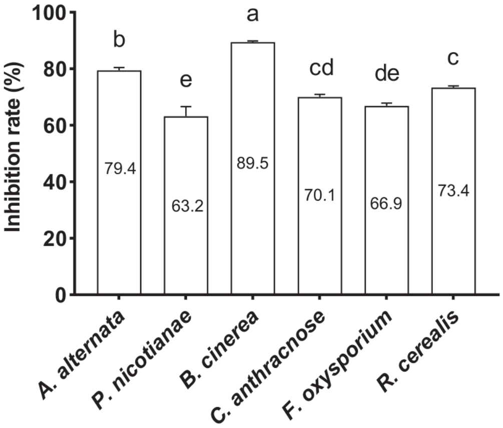

Inhibition rate (%)
100
80
60
40
20
0
b
e
a
cd
de
c
79.4
63.2
89.5
70.1
66.9
73.4
A. alternata
P. nicotianae
B. cinerea
C. anthracnose
F. oxysporium
R. cerealis

Figure S2

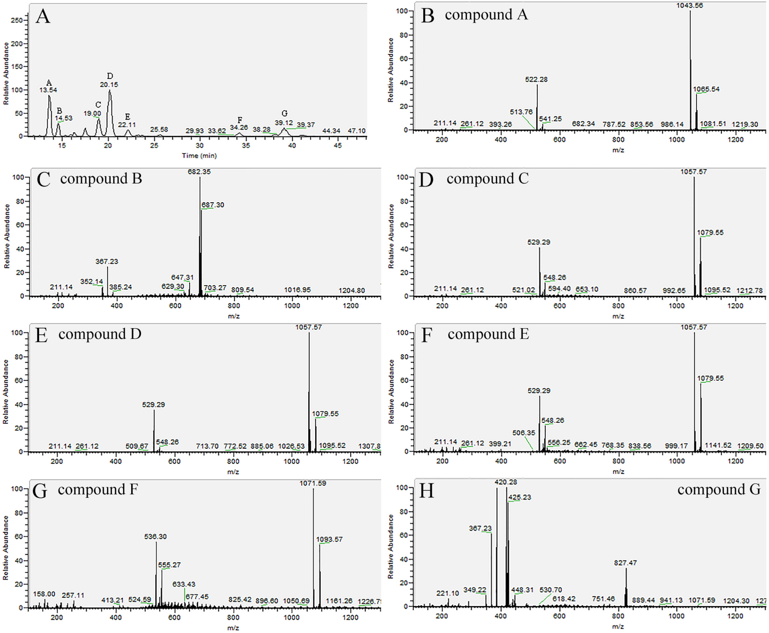
A
Relative Abundance
Time (min)
A 13.54
B 14.53
C 19.00
D 20.15
E 22.11
F 34.26
G 39.12
B compound A
1043.56
1065.54
522.28
m/z
C compound B
682.35
687.30
367.23
D compound C
1057.57
1079.55
529.29
E compound D
1057.57
1079.55
529.29
F compound E
1057.57
1079.55
529.29
G compound F
1071.59
1093.57
536.30
H compound G
420.28
425.23
367.23
827.47

Figure S3

A

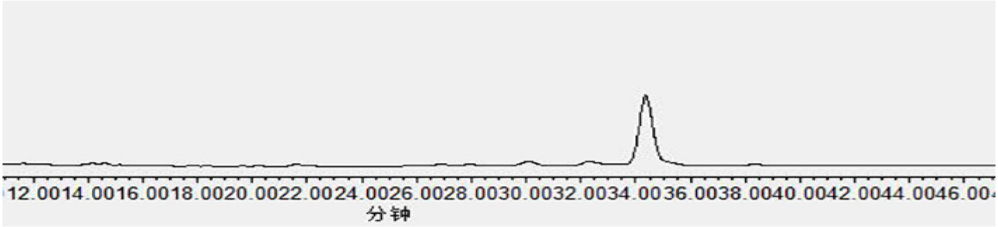

B

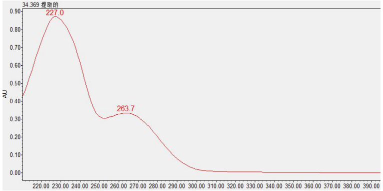

C

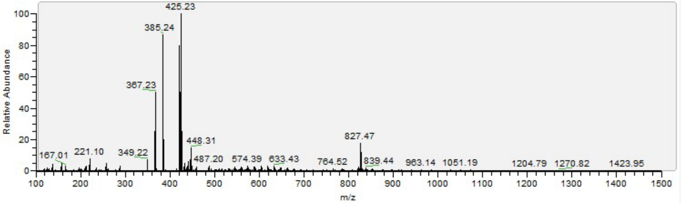

Figure S4

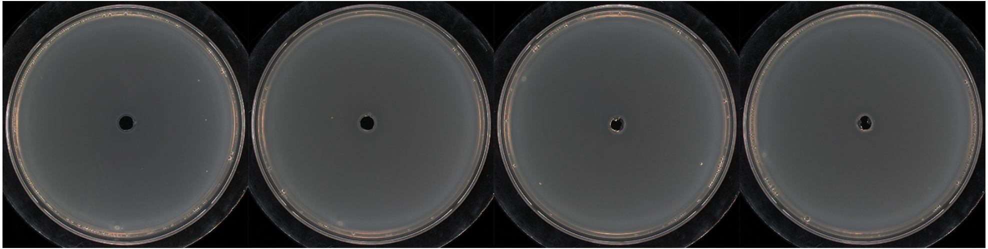

CK (Methanol) | Iturin A standard (10 mg/mL) | Iturin A standard (5 mg/mL) | Iturin A standard (1 mg/mL)

Figure S5

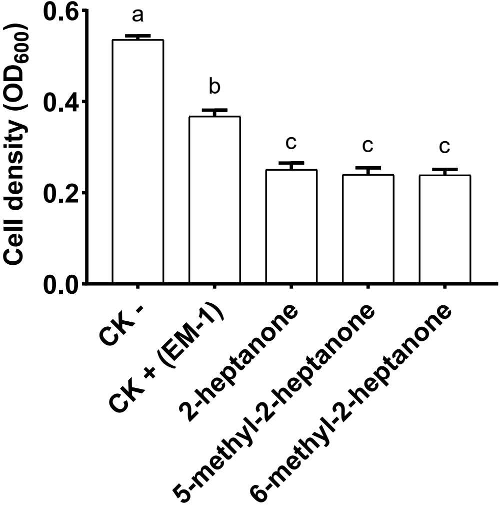

a
b
c
c
c
0.6
0.4
0.2
0.0
Cell density (OD_{600})
CK -
CK + (EM-1)
2-heptanone
5-methyl-2-heptanone
6-methyl-2-heptanone

Figure S6

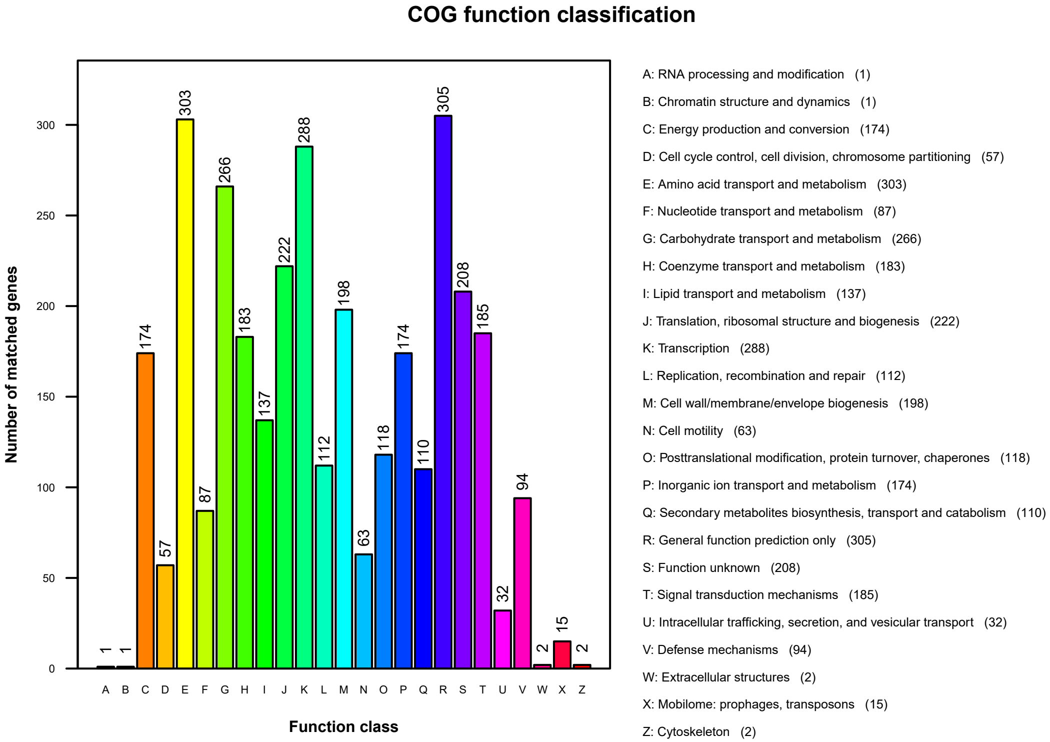
COG function classification
Number of matched genes
Function class
0
50
100
150
200
250
300
1
1
174
57
303
87
266
183
137
222
288
112
198
63
118
174
110
305
208
185
32
94
2
15
2
A B C D E F G H I J K L M N O P Q R S T U V W X Z
A: RNA processing and modification (1)
B: Chromatin structure and dynamics (1)
C: Energy production and conversion (174)
D: Cell cycle control, cell division, chromosome partitioning (57)
E: Amino acid transport and metabolism (303)
F: Nucleotide transport and metabolism (87)
G: Carbohydrate transport and metabolism (266)
H: Coenzyme transport and metabolism (183)
I: Lipid transport and metabolism (137)
J: Translation, ribosomal structure and biogenesis (222)
K: Transcription (288)
L: Replication, recombination and repair (112)
M: Cell wall/membrane/envelope biogenesis (198)
N: Cell motility (63)
O: Posttranslational modification, protein turnover, chaperones (118)
P: Inorganic ion transport and metabolism (174)
Q: Secondary metabolites biosynthesis, transport and catabolism (110)
R: General function prediction only (305)
S: Function unknown (208)
T: Signal transduction mechanisms (185)
U: Intracellular trafficking, secretion, and vesicular transport (32)
V: Defense mechanisms (94)
W: Extracellular structures (2)
X: Mobilome: prophages, transposons (15)
Z: Cytoskeleton (2)
